# Characterization of Microvesicles in Septic Shock Using High-Sensitivity Flow Cytometry

**DOI:** 10.1186/2197-425X-3-S1-A517

**Published:** 2015-10-01

**Authors:** GF Lehner, U Harler, C Feistritzer, J Hasslacher, S Dunzendorfer, R Bellmann, M Joannidis

**Affiliations:** Medical University of Innsbruck, Department of Internal Medicine - Division of Intensive Care and Emergency Medicine, Innsbruck, Austria; Medical University of Innsbruck, Department of Internal Medicine - Haematology and Oncology, Innsbruck, Austria

## Introduction

Endothelial dysfunction, activation and apoptosis are considered key features of sepsis. Microvesicles (MV) can be released by cells either constitutively, upon stimulation or apoptosis. Since endothelial derived microvesicles (EMV) were found to be elevated in diseases associated with endothelial activation and damage [1] they seem to be a promising diagnostic tool to assess the state of the endothelium.

## Objectives

This study was aimed at testing the hypothesis that patients in early phase of septic shock have high levels of EMV circulating in blood.

## Methods

Platelet free plasma was prepared from 18 healthy volunteers as well as 30 patients with septic shock treated in a medical intensive care unit and analyzed by high-sensitivity flow cytometry which has a high sensitivity for small and previously undetectable MV. Results were controlled for potential artifacts and absolute counts of different MV subtypes were compared between groups by using the Mann-Whitney-U test. Patients with an International Society of Thrombosis and Haemostasis (ISTH) - disseminated intravascular coagulation (DIC) score of at least five were classified as having an overt DIC. Results are presented as median and interquartile range.

## Results

Absolute counts of CD144+, CD62E+ and CD106+ MV, specific for endothelial-derived MV, were low in patients with septic shock as well as in healthy volunteers. Patients with septic shock had higher levels of apparently predominantly leukocyte-derived CD31+/CD41- MV (58.48 (74.78) vs. 19.47 (12.64) MV/µl; p < 0.001) compared to healthy volunteers. Patients who died within 48 hours after inclusion did show significantly elevated CD31+ MV (599.67 (674.69) vs. 239.24 (284.81) MV/µl;p = 0.015) and CD41+ MV (639.14 (648.40) vs. 221.54 (337.35) MV/µl; p = 0.037), indicating release by excessive platelet activation. Patients dying within 48 hours had significantly higher levels of CD31+/CD41-/AnnexinV- MV (51.86 (234.89) vs. 18.89 (21.35) MV/µl; p = 0.028), probably released by activated leukocytes.

## Conclusions

EMV considered as a manifestation of endothelial dysfunction in sepsis are not elevated in the majority of patients. Patients with septic shock who die within 48 hours exhibit features of excessive leukocyte- and platelet-activation.

## Grant Acknowledgment

Austrian Nationalbank
